# EPA Proposes Tighter Particulate Air Pollution Standards

**DOI:** 10.1289/ehp.120-a348a

**Published:** 2012-08-31

**Authors:** Bob Weinhold

**Affiliations:** Bob Weinhold, MA, has covered environmental health issues for numerous outlets since 1996. He is a member of the Society of Environmental Journalists.

Particulate matter (PM) is one of six criteria pollutants regulated by National Ambient Air Quality Standards (NAAQS). These standards are twofold: a primary standard protects human health, and a secondary standard protects crops, ecosystems, and other forms of “public welfare.” The U.S. Environmental Protection Agency (EPA) last revisited the PM standards in 2006. Now, in response to a court order mandating action on the overdue review of these rules, the agency has proposed a stricter set of new standards.^1^

PM can be emitted directly from sources such as vehicles, power plants, burning biomass, and various industrial operations, or it can form as a reaction product. PM can contribute to a wide range of adverse health effects in people, with effects varying with the size and composition of the particles. Health damage occurs even in localities that meet current PM standards;^2^ the EPA’s advisory panel of independent experts, the Clean Air Scientific Advisory Committee (CASAC), noted in its correspondence with the agency regarding the proposed rules that “Although there is increasing uncertainty at lower levels [of PM exposure], there is no evidence of . . . a level below which there is no risk for adverse health effects.”^3^

For long-term effects of fine PM (PM_2.5_ ), CASAC recommended the primary health standard be tightened from a current annual average of 15 µg/m^3^ to somewhere in the range of 11–13 µg/m^3^.^3^ The EPA is proposing a standard in the range of 12–13 µg/m^3^ and is accepting public comments on levels down to 11 µg/m^3^. To address short-term effects, CASAC recommended a range of 30–35 µg/m^3^ averaged over 24 hours; the agency proposes to retain the current standard of 35 µg/m^3^.

**Figure:**
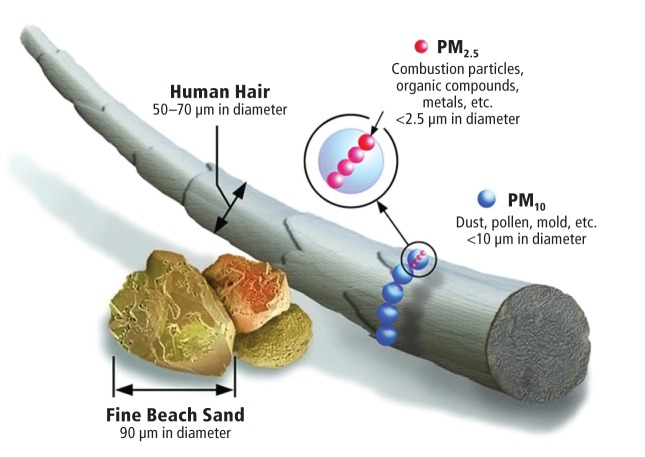
Particle size is an important factor in how PM affects human health. Larger PM_10_ is deposited mostly in the nose and throat. Because PM_2.5_ can penetrate much deeper into the lung, it poses a greater health threat. Image courtesy of U.S. EPA

For coarse PM (PM_10_), the CASAC recommended the agency change not just the level of the standard but also its “form”—the air quality statistics used to determine whether an area is in compliance. The committee recommended adopting a level of 65–75 µg/m^3^ as the 98th percentile 24-hour concentration averaged over three years. The agency is proposing to keep the standard at the current 150 µg/m^3^ based on a so-called one-expected exceedance form—the 24-hour limit is not to be exceeded more than once a year averaged over three years.

The agency estimates that at any point in the proposed ranges the dollars saved from avoided health costs, sick days, and deaths would far outweigh costs paid by affected states, tribal lands, and counties to achieve the lower standards.^4^ With PM_2.5_ standards of 13 µg/m^3^ (annual) and 35 µg/m^3^ (24-hour), the EPA calculates annual health benefits of $88–220 million, with costs of $2.9 million.^5^ Substituting an annual standard of 12 µg/m^3^, annual health benefits are estimated at $2.3–5.9 billion, with implementation costs of $69 million. At an annual standard of 11 µg/m^3^, annual health benefits would be an estimated $9.2–23.0 billion, with costs of $270 million.

The agency also calculated a scenario with an annual standard of 11 µg/m^3^ and a 24-hour standard of 30 µg/m^3^. Both the benefits and implementation costs are estimated to be roughly 50% higher than the configuration of 11 µg/m^3^ (annual) and 35 µg/m^3^ (24-hour).

About 30% of the U.S. population lives in the 191 counties or parts of counties designated as “nonattainment” for the current annual PM_2.5_ standard. Attainment status is based on a rolling three years’ worth of PM data for those counties with air monitors; for the rest, state and EPA officials must estimate each county’s contribution to the larger area’s PM pollution.

In figures published with the proposed standards, the EPA estimated 33 counties with monitors (with total populations of more than 27 million) would violate an annual standard of 13 µg/m^3^, an additional 49 counties (with more than 27 million additional people) would violate 12 µg/m^3^, and an additional 86 counties (with tens of millions more people) would violate 11 µg/m^3^.^6^ These figures were based on 2008–2010 monitoring data.

Ted Cromwell, senior principal for environmental policy at the National Rural Electric Cooperative Association, questions the wisdom of further tightening the standards without knowing which specific chemical constituents of PM_2.5_ are responsible for associated health effects.^7^ He’d prefer to continue implementation of the current standard until research more definitively pins down those substances, and then target them specifically.

The EPA is reviewing public comments on the proposal and is required by the court-approved consent decree to issue final rules by 14 December 2012. Mitigation measures are supposed to begin by 2015 and must be fully implemented by 2020.
